# Multi-slice ptychography enables high-resolution measurements in extended chemical reactors

**DOI:** 10.1038/s41598-020-80926-6

**Published:** 2021-01-15

**Authors:** Maik Kahnt, Lukas Grote, Dennis Brückner, Martin Seyrich, Felix Wittwer, Dorota Koziej, Christian G. Schroer

**Affiliations:** 1 Deutsches Elektronen-Synchrotron DESY, Notkestraße 85, 22607 Hamburg, Germany; 2 MAX IV Laboratory, Lund University, Fotongatan 2, 224 84 Lund, Sweden; 3 Institute for Nanostructure and Solid State Physics, Center for Hybrid Nanostructures (CHyN), Universität Hamburg, Luruper Chaussee 149, Building 600, 22761 Hamburg, Germany; 4 Department Physik, Universität Hamburg, Luruper Chaussee 149, 22761 Hamburg, Germany

**Keywords:** Optical physics, X-rays, Imaging techniques, Microscopy

## Abstract

Ptychographic X-ray microscopy is an ideal tool to observe chemical processes under in situ conditions. Chemical reactors, however, are often thicker than the depth of field, limiting the lateral spatial resolution in projection images. To overcome this limit and reach higher lateral spatial resolution, wave propagation within the sample environment has to be taken into account. Here, we demonstrate this effect recording a ptychographic projection of copper(I) oxide nanocubes grown on two sides of a polyimide foil. Reconstructing the nanocubes using the conventional ptychographic model shows the limitation in the achieved resolution due to the thickness of the foil. Whereas, utilizing a multi-slice approach unambiguously separates two sharper reconstructions of nanocubes on both sides of the foil. Moreover, we illustrate how ptychographic multi-slice reconstructions are crucial for high-quality imaging of chemical processes by ex situ studying copper(I) oxide nanocubes grown on the walls of a liquid cell.

## Introduction

The large penetration depth of hard X-rays in matter makes them an attractive probe to study the inner structures of objects without the need for destructive sample preparation. In particular, X-rays can penetrate sample environments and chemical reactors, making in situ and *operando* studies of physical and chemical processes feasible^1–10^. X-ray microscopy is well suited to measure chemical and physical properties with high spatial resolution, even in three dimensions, if it is combined with tomographic techniques.

As a scanning coherent X-ray diffraction imaging technique, X-ray ptychography^11^ takes full advantage of the high brightness of synchrotron radiation sources and greatly benefits from the latest generation of ultra-low emittance sources^12–14^. Ptychography has revolutionised X-ray microscopy and is routinely used at synchrotron radiation sources around the world^11^. Compared to conventional X-ray full-field microscopy, X-ray ptychography offers superior resolution, reaching into the single-digit nanometer range^15,16^. X-ray ptychography is well suited for in situ and *operando* measurements and has been utilized to study various physical and chemical processes^17–20^. In all cases, the sample is enclosed inside some sample environment that is penetrated by the X-rays during the measurement. For ptychography to work in the thin object approximation, the sample and its surrounding container have to be optically thin. While this can be achieved by using microreactors, the latter are not suited for all types of processes, as some processes cannot be downscaled. For larger reactors, the sample environment may be too thick along the optical axis and exceed the depth of field of the conventional ptychographic model. Such sample environments call for a ptychographic model with extended depth of field, the multi-slice model^21–30^.

**Figure 1:**
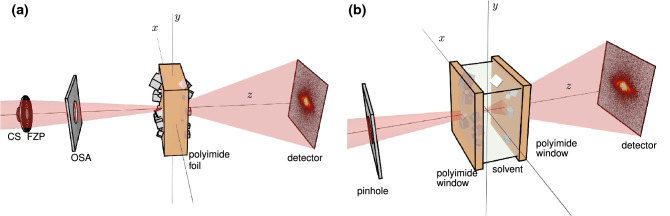
Schemes of the two experiments: (**a**) the ptychographic imaging experiment on a single polyimide foil: The central stop (CS), the Fresenel zone plate (FZP) and the order sorting aperture (OSA) are used to focus the coherent X-ray beam (red) on the polyimide foil with copper(I) oxide nanocubes deposited on both sides. (**b**) the ptychographic imaging experiment on the chemical reactor: the X-ray beam is focussed by a set of two 1D-focussing lenses (not shown), cleaned up by the pinhole, passes through the extended chemical reactor with particles on the downstream side of the upstream window and the upstream side of the downstream window and is finally measured in the far field by a photon counting pixel detector. In both experiments the sample is scanned perpendicularly to the beam (in *x* and *y* direction), while diffraction patterns are recorded in the far field downstream of the sample by a photon counting pixel detector. The images were created with *blender*^34^ (version 2.83) and were composed using *matplotlib*^35^ (version 3.1.3^36^).

A model system to demonstrate the need for multi-slice imaging is a polyimide foil with copper(I) oxide nanocubes deposited on both of its sides. To nucleate the copper(I) oxide on both sides, the foil was immersed in the reaction solution during the non-aqueous nucleation and growth process. The polyimide foil was then removed from the reactor with the nanocubes firmly attached to both its surfaces. For a second demonstration, we mimic an in situ experiment by studying the same copper(I) oxide nanocubes grown on the two inner surfaces of the polyimide windows of a chemical reactor (see Fig. 1b).

**Figure 2:**
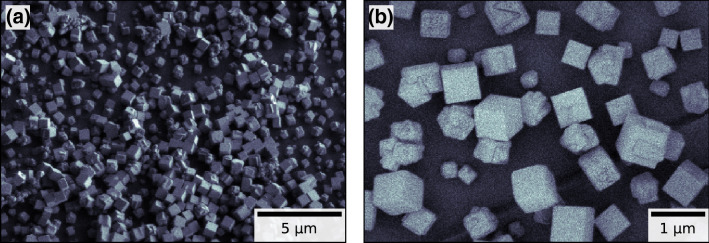
Scanning electron microscopy (SEM) overview images of two different regions on the same side of a polyimide foil covered with copper(I) oxide nanocubes similar to the ones used in the X-ray imaging experiment. (**a**) Secondary electron image of the tilted sample taken at ${1}\,\hbox {keV}$ accelerating voltage showing mostly smooth nanocubes and varying local covering densities of the surface with nanocubes; (**b**) Mixed secondary and back scattered electron beam image taken at ${0.3}\,\hbox {keV}$ accelerating voltage reveals irregularly facets at the surface of some nanocubes and additionally irregular shaped particles. The images were recorded using a *Hitachi Regulus 8200 Series FE-SEM*, operation on the control software version 3.2. The figure was created using *matplotlib*^35^ (version 3.1.3^36^).

The copper(I) oxide nanocubes are formed via a non-aqueous route based on a metal-organic precursor dissolved in benzyl alcohol^31^. The solution is heated, which introduces a heterogeneous nucleation of cubic nanoparticles at solid surfaces in contact with the liquid^32,33^. Solid surfaces can be the walls of the reaction container or an additional piece of polyimide foil submerged in the solution. The particles nucleate on the surface and grow to a maximum size that depends on several reaction conditions such as the specific precursor, the precursor concentration or the temperature during the growth process. At a reaction temperature of 180 °C, the particles grew to a maximum size of about 500 nm (see Fig. 2). Like many chemical reactions, this deposition occurs only above a certain process volume due to a disturbance of the reaction kinetics upon downscaling. The slow deposition and growth of the nanocubes is reaction-controlled, meaning that the diffusion of new material towards the particles is fast compared to the consumption of material upon particle growth. Here, the assumption is that above the surface where the particles grow, there is a sufficiently vast continuum of benzyl alcohol in which the concentration of the dissolved metal-organic precursor never changes significantly in the vicinity of the particles upon precursor consumption during the reaction. Reducing the size of the reaction chamber even in one dimension along the beam axis would limit the diffusion of precursor material, which could make the process diffusion-controlled and alter the reaction product. The smallest feasible size for reactors that ensure reaction-controlled particle growth is on the order of half a millimetre, well beyond the depth of field for conventional high-resolution X-ray ptychography. To prove the feasibility of an in situ experiment, we first image the copper(I) oxide nanocubes deposited on the two sides of a polyimide foil (see Fig. 1a) and then on the polyimide entry and exit windows of the chemical reactor (see Fig. 1b).

The polyimide foil had a thickness of $\approx {100}\,{\upmu \hbox {m}}$, which is already smaller than the minimal extension of the reactor along the optical axis. For high-resolution ptychography, even a thickness on the order of $\approx {100}\,{\upmu \hbox {m}}$ exceeds the depth of field, demonstrating the need for multi-slice reconstructions for the high-resolution in situ X-ray imaging study of the reaction.

## Results

In the conventional ptychographical model, the sample is assumed to be thin, allowing the sample to be modeled as a two-dimensional complex-valued field $O\left( {\mathbf {r}}\right)$ and the interaction with a probing coherent wavefront $P\left( {\mathbf {r}}\right)$ can be described by the multiplication of the two^37,38^.

There are three thickness regimes to differentiate.

First: the sample is so thick that the X-ray beam changes significantly while propagating through the sample and thus the sample cannot be regarded as optically thin. For the first experiment described here, the depth of field of the Fresnel zone plate that was used for focusing the X-rays was calculated to be $\text{ DOF}_{\text{ FZP }} = {143.86}\,{\upmu \hbox {m}}$, which is larger than the sample thickness of ${100}\,{\upmu \hbox {m}}$. Thus the first experiment does not fall into this category. This would change, if the numerical aperture of the optics was increased, e. g., to reach higher spatial resolution in conventional scanning microscopy, or if the sample would be even thicker, like in the second presented experiment. There the depth of field of the nano-focussing lenses was estimated to be $\text{ DOF}_{\text{ NFL }} = {182.95}\,{\upmu \hbox {m}}$ (see “Methods” section for details), which is smaller than the estimated sample thickness of ${650}\,{\upmu \hbox {m}}$. The chemical reactor can thus not be modeled as a thin sample in the presented experiment.

Second: the sample is thinner than the depth of field of the illuminating beam and the latter does not significantly change along the whole thickness of the sample. In ptychographic imaging, however, the largest scattering angle with sufficient scattered signal on the detector defines the effective numerical aperture of the virtual objective lens. It often exceeds that of the illuminating beam significantly and thus allows for far higher resolution than the illuminating beam size. Along with this comes a reduced depth of field for ptychography that is smaller than that of the illuminating beam and can be smaller than the thickness of the sample. If it really becomes smaller than the thickness of the sample, the sample has to be regarded as optically thick for ptychography. In the presented experiments, the smallest possible depth of field for ptychographic imaging is defined by the physical size of the cropped diffraction patterns on the detector. For the experiment on the polyimide foil it is calculated to be ${6.39}\,{\upmu \hbox {m}}$ and for the experiment on the chemical reactor it is calculated to be ${2.58}\,{\upmu \hbox {m}}$ (see “Methods” section for details). The copper(I) oxide nanocubes themselves are therefore thin enough to be modeled as thin samples in both experiments. The polyimide foil and the extended chemical reactor however are much thicker than those two numbers respectively. For the latter it was already established that it can not be modeled as a thin sample. For the experiment on the polyimide foil, this case of being thin for the probing X-ray beam but not being also necessarily thin for the virtual imaging lens, does apply. To determine if the foil can be considered as optically thick or optically thin, the effectively used numerical aperture of the detector has to be determined. It can be smaller than the chosen cropping of the diffraction patters, thus resulting in an effective depth of field larger than the minimal achievable depth of fields calculated above.

Third: the sample is thinner than the depth of field of the ptychographic imaging geometry. In that case, the sample is optically thin and the conventional ptychographic model can be applied. This would for example hold true for the nanoparticles on one side of the polyimide foil and the particles on one window of the chemical reactor.

To resolve the question if the experimental data taken on the polyimide foil can be regarded as optically thin, the previous arguments can be turned around, asking what spatial resolution can be expected if one assumes a given object to be optically thin. In that case a depth of field matching the sample thickness implies a lower limit for the spatial resolution. This has been investigated by Tsai et al.^25^, where this relationship has been determined numerically:

1 $$\begin{aligned} T \le 5.2 \cdot \frac{\left( \delta r\right) ^2}{\lambda }, \end{aligned}$$

where *T* is the thickness of a sample that is treated as thin, $\delta r$ is the achievable image resolution and $\lambda$ is the wavelength of the probing X-ray beam. Using the experimental parameters for the experiment on the polyimide foil (see “Methods” section for details), the known sample thickness of $T = {100}\,{\upmu \hbox {m}}$, Eq. (1) yields a resolution $\delta r$ of at best ${51.1}\,\hbox {nm}$ in thin-sample approximation.

**Figure 3:**
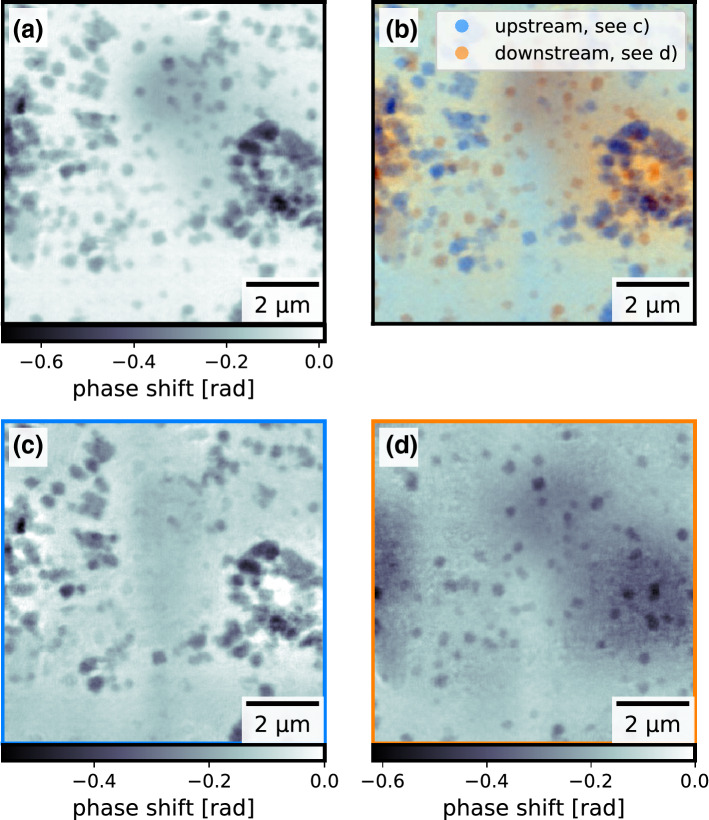
Phases of the reconstructed objects from the single polyimide foil experiment in direct comparison: (**a**) single object slice reconstruction using ePIE (**b**) colored overlay of the upstream object slice (blue, **c**) and the downstream object slice (orange, **d**), reconstructed using 3PIE. The figure was created using *matplotlib*^35^ (version 3.1.3^36^).

**Figure 4:**
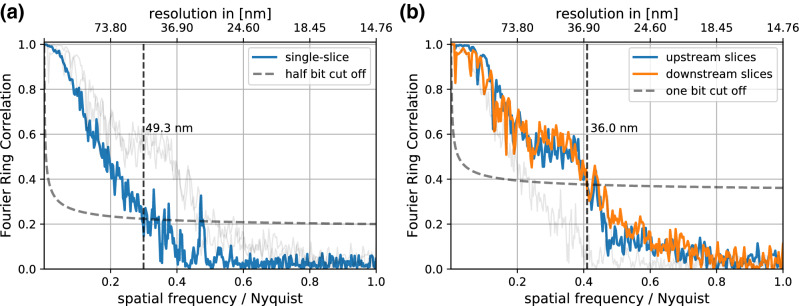
Estimated resolutions of the single polyimide foil experiment using Fourier ring correlation: (**a**) using two single slice reconstructions from halved datasets (**b**) using the two multi-slice reconstructions with inverted initialization order. The light gray lines show the FRC of the respective other reconstructions for an easier comparison. The figure was created using *matplotlib*^35^ (version 3.1.3^36^).

To be able to compare this resolution limit with the actually achieved resolution, the recorded data from the experiment on the polyimide foil needed to be reconstructed using the thin sample approximation. We separated the set of diffraction patterns into two halves and reconstructed both halves with identical reconstruction parameters using the extended ptychographic iterative engine (ePIE) algorithm^38^, which is based on the thin-sample approximation (see “Methods” section for details on the reconstructions). One of the reconstructed objects is shown in Fig. 3a, showing the copper(I) oxide nanocubes scattered all over the imaged field of view. We estimated the resolution using Fourier ring correlation (FRC)^39,40^ between the two reconstructions to be ${49.3}\,\hbox {nm}$ (see Fig. 4a). In this experiment the resolution in the ptychographic image thus might have been limited by the depth of field as described in by Eq. (1). If that were the case, the object needs to be modeled as optically thick to push the resolution beyond this limit.

To test this hypothesis, multi-slice reconstructions (MSR) of the same dataset were carried out subsequently using the 3PIE algorithm^21^, modeling the sample as two distinct slices separated by the measured ${100}\,{\upmu \hbox {m}}$ thickness of the polyimide foil (see “Methods” section for details on the reconstructions). The reconstructed object slices are shown in Fig. 3b–d. We estimated a resolution of ${36}\,\hbox {nm}$ in all reconstructed slices by comparing the reconstructed slices from the two reconstructions using FRC (see Fig. 4b). The improvement in resolution by accounting for an X-ray optically thick sample confirms the consideration above.

**Figure 5:**
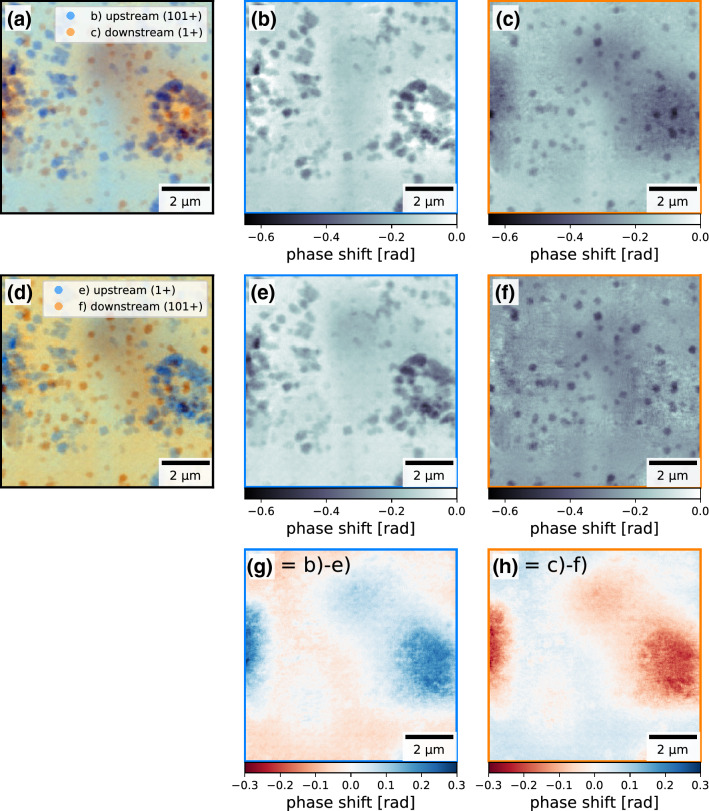
Comparison of two multi-slice reconstructions of the same recorded dataset from the single polyimide foil experiment with inverted initialization order of the two object slices: (**a**)–(**c**) reconstructed object slices where the downstream sample slice was initialized first, (**d**)–(**f**) reconstructed object slices where the upstream sample slice was initialized first, (**g**) and (**h**) difference between the reconstructed up-/downstream slices respectively between the two initialization cases showing the long range cloud like artefacts switching slices depending on the initialization order. The figure was created using *matplotlib*^35^ (version 3.1.3^36^).

Both the ePIE and the 3PIE algorithm reconstruct the same particles at the same position. Every single copper(I) oxide nanocube can be found either in the upstream sample slice or the downstream sample slice of the multi-slice reconstruction. None of the copper(I) oxide nanocubes is reconstructed in both slices. The algorithm unambiguously separated the particles according to which surface they are located on. Long-ranged artefacts (see Fig. 5g,h) can be seen in the background. As they are the same in the single slice reconstruction and the multi-slice reconstruction, we can exclude that the introduction of a second object slice and the connected reduced redundancy in the data is the reason for their appearance. The reconstruction of these low spatial frequencies is a known problem of such phase retrieval algorithms in the presence of short-term instabilities^41^.

Looking at the background behind the particles, a vertical stripe can be seen in the center of both multi-slice reconstructed object slices. In the upstream slice (see Fig. 3c) it appears more phase shifting than its surrounding and in the downstream slice (see Fig. 3d) it appears less phase shifting than its surrounding. Adding both slices together makes this feature vanish and results in an image very similar to the single slice reconstruction (see Fig. 3a). There is no way of disproving that those are real features in both the upstream slice and the downstream slice. However, the fact that these two stripes cancel out so perfectly suggest that this is another artefact introduced by the 3PIE reconstruction and not real features of the sample. As the stripe is vertical and thus follows the fast scanning direction, we believe this artefact is the result of instability of the probing X-ray beam, as this is more likely than two features in the two slices aligning and cancelling out perfectly.

The two sides of the polyimide foil show a distinct difference of copper(I) oxide nanocube coverage. These local differences in covering density of particles have already been observed between different regions on the same side of the polyimide foil using scanning electron microscopy (see Fig. 2). Therefore, the differences in covering density between the two reconstructed slices do not necessarily indicate global differences between the two sides of the polyimide foil, but rather only local differences in the reconstructed field of view. That is why the differences in coverage do not indicate an arbitrary separation of the copper(I) oxide nanocubes to one of the reconstructed slices. In fact, the multi-slice reconstruction of a second scan resulted in the very same separation of particles.

Changing the order of initialization (see “Methods” section for details) of the slices in the algorithm resulted in the very same separation of the particles between the two slices (see Fig. 5). Only the long-range artefacts switched slices, as they always remained in the slice which was initialized first (see Fig. 5g,h). This strengthens our confidence in the truthfulness of the separation into particles on the upstream surface and particles on the downstream surface of the polyimide foil. However, because of the small size of the scanned area ($10 \times {10\,}\upmu \hbox {m}^2$) compared to the size of the full sample ($10 \times {10}\hbox { mm}^{2}$) it was not possible to independently verify the separation of particles into upstream and downstream surface after the X-ray experiments using SEM imaging.

**Figure 6:**
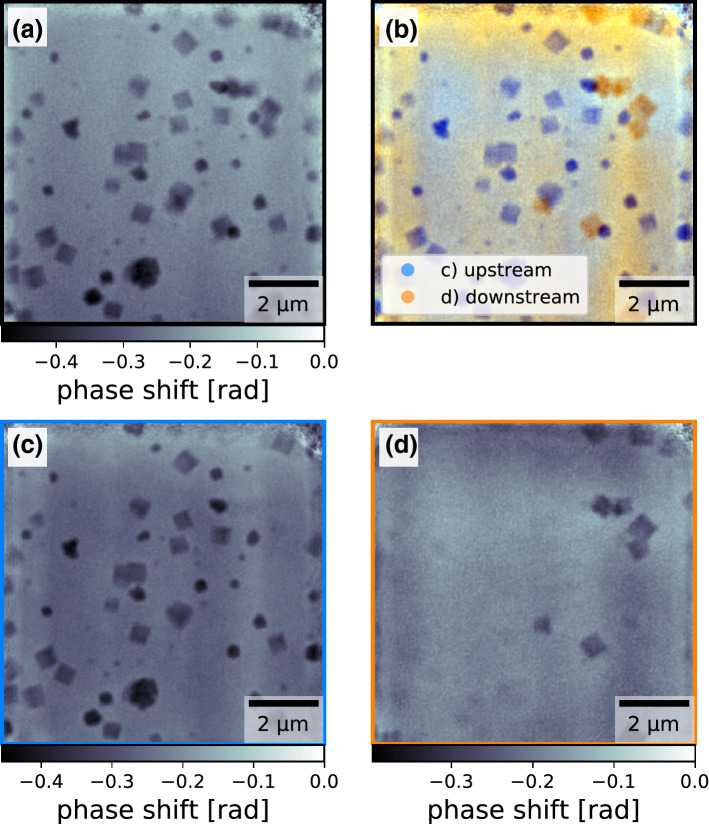
Phase reconstructions of the experiment using the chemical reactor: (**a**) ptychographically reconstructed object phase using the single slice ePIE algorithm (**b**) colored overlay of the upstream object slice (blue, **c**) and the downstream object slice (orange, **d**), reconstructed using the multi slice 3PIE algorithm. The figure was created using *matplotlib*^35^ (version 3.1.3^36^).

The final step towards an in situ experiment was to image particles inside the reaction chamber they were grown in. A chemical reactor with two polyimide foils as entry and exit windows for the probing X-ray beam was designed and built. In a first test, copper(I) oxide nanocubes were grown inside the chemical reactor in the lab without a probing X-ray beam. The precursor solution was removed from the chemical reactor, the chemical reactor was disassembled and both the entry and exit windows were imaged by SEM to verify that nanocubes have indeed grown on both windows. Afterwards the chemical reactor was reassembled using the very same windows and filled with the solvent. The distance between the inside window surfaces was estimated to be around ${650}\,{\upmu \hbox {m}}$, which is significantly more than the previous test based on the ${100}\,{\upmu \hbox {m}}$ thick polyimide foil. The reassembled chemical reactor was then ptychographically measured using X-rays without heating it (see Fig. 6a) to verify that the particles can be imaged in this sample environment (experimental details can be found in the “Methods” section). The X-ray focus was created by two sets of 1D nano-focussing lenses^42^ and cleaned by a pinhole between the lenses and the sample, resulting in a depth of field of approximately ${183}\,{\upmu \hbox {m}}$, which is significantly smaller than the separation of the inner surfaces of the entry window and exit window. Hence, the thin sample approximation was not applicable for this experimental situation either. As with the single foil, both the single-slice reconstruction using the ePIE algorithm (see Fig. 6a) and the multi-slice reconstruction using the 3PIE algorithm (see Fig. 6b–d) succeeded and the copper(I) oxide nanocubes could be clearly resolved. Again the multi-slice reconstructions appeared sharper and separated particles on the upstream window and on the downstream window, again revealing difference in local covering density of particles.

## Discussion

We have shown that the copper(I) oxide nanocubes themselves are thinner than the maximal depth resolution achievable with the given detector size and thus justify a single-slice reconstruction using the ePIE algorithm. However, once they are present on two surfaces, separated by a distance *T*, a conventional thin-object reconstruction, e. g., by ePIE, is limited in spatial resolution according to Eq. (1). For the example with the nanocubes grown on the two sides of the foil, this limit is ${51.1}\,\hbox {nm}$. In order to reach higher spatial resolution, a multi-slice model of the sample needs to be employed. The reconstruction using a multi-slice algorithm allows to separate the imaged nanocubes reliably according to which surface of the polyimide foil they grew on.

We have furthermore proven that these nanocubes can be imaged inside the (cold) chemical reactor they were grown in, where the separation of the upstream surface and downstream surface covered in particles was even larger than with the previous experiment on the single polyimide foil. Here, the multi-slice reconstruction allowed again to separate the imaged nanocubes reliably according to which polyimide foil window they grew on.

High-resolution in situ X-ray imaging measurements following the presented growth process can not be performed in a way that a thin-sample approximation is applicable. The X-ray beam will always have to pass through at least two container surfaces which are covered with copper(I) oxide nanocubes. Those two surfaces will have to be spaced far enough apart to accommodate the minimum volume of liquid needed for this reaction to occur in the first place. This distance between the two container surfaces is limiting the achievable resolution in the reconstructions, if a reconstruction algorithm based on the thin sample approximation is used.

Ptychographic multi-slice reconstructions allow to image the growth process of these copper(I) oxide nanocubes as it is and without imposing experimental restrictions on the imaged sample being optically thin. The separation of the nanocubes between the two layers is conclusive, because it does not depend on the chosen initialization order of the slices and is identical over multiple measurements.

## Methods

### Sample preparation: single polyimide foil

To prepare the sample of copper(I) oxide nanocubes on a support, ${0.25}\,\hbox {mmol}$ (${65}\hbox { mg}$) of copper acetylacetonate (Cu(acac)$_2$, $98\,\%$, Acros Organics) were mixed with ${5}\,\hbox {mL}$ of benzyl alcohol (puriss., Sigma Aldrich) under ambient conditions and stirred for 15 min^33^. A piece of polyimide foil (Kapton HN, DuPont, USA) with a size of approximately ${10}\,\hbox {mm} \times {10}\,\hbox {mm}$ was cleaned by rinsing with ethanol (abs., Scharlau). ${0.8}\,\hbox {mL}$ of the Cu(acac)$_2$ solution were transferred into a specially designed PEEK reaction vial^43^ with a total volume of ${1}\,\hbox {mL}$ and the polyimide foil was placed upright into the vial in a way that both sides were in contact with the precursor solution. After sealing the vial with a PEEK cap and a PTFE sealing ring, it was clamped into a heatable brass chassis. The vial was heated to ${180}\,^{\circ }\hbox {C}$ with a rate of 1 °C min^−1^ and kept at that temperature for ${60}\,\hbox {min}$, followed by cooling down quickly. The polyimide foil was cleaned again by rinsing with ethanol and dried at ${60}\,^{\circ }\hbox {C}$ overnight. All chemicals were used as purchased without further purification.

### Experiment: single polyimide foil

The experiment was performed at the hard X-ray nanoprobe station PtyNAMi of beamline P06 at the synchrotron radiation source PETRA III (Hamburg, Germany)^44,45^. The polyimide foil with the copper(I) oxide particles on both sides was glued to a thin silicon frame, covering a hole of ${10}\,\hbox {mm}$ diameter. The plate with the foil on top was clamped into a sample holder, which was placed inside the PtyNAMi setup^46,47^. The X-ray beam coming from the undulator was monochromatized to ${9.1}\,\hbox {keV}$ using a Si-(111) double-crystal monochromator. Higher harmonics were suppressed by a pair of flat horizontally deflecting mirrors. A Fresnel zone plate (FZP) with ${125}\,{\upmu \hbox {m}}$ diameter and ${70}\,\hbox {nm}$ outer-most zone width was used to focus the X-ray beam. The focal length of the FZP was ${64.2}\,\hbox {mm}$. Therefore, the depth of field of the Fresnel zone plate is ${143.86}\,{\upmu \hbox {m}}$.

2 $$\begin{aligned} \text{ DOF}_{ \text{ FZP }} = \frac{\lambda }{\text {NA}^2_{\text{ FZP }}} = \frac{4 \cdot \lambda f^2_{\text{ FZP }}}{ D^2_{\text{ FZP }}} = \frac{4 \cdot {0.136}\,\hbox {nm} \cdot \left( {64.2}\,\hbox {mm}\right) ^2}{\left( {0.125}\,\hbox {mm}\right) ^2} = {143.86}\,{\upmu \hbox {m}} \end{aligned}$$

The sample was placed approximately ${750}\,{\upmu \hbox {m}}$ upstream of the focus, resulting in a beam diameter of ${1.4}\,{\upmu \hbox {m}}$ (FWHM) at the sample position. Piezoelectric motors were used to scan the sample on a rectangular grid with a size of ${10}\,{\upmu \hbox {m}} \times {10}\,{\upmu \hbox {m}}$ in $20 \times 20$ steps. At each of the $21 \times 21 = 441$ positions, a far-field diffraction pattern was recorded with ${1} \hbox {s}$ exposure time using an EIGER X 4M detector (DECTRIS, Switzerland, ${75}\,{\upmu \hbox {m}}$ pixel size) positioned ${4.16}\,\hbox {m}$ downstream of the sample. The relative sample positions were measured by three interferometers retro-reflected by a ball-lens located below the sample^46^. The thickness of the polyimide foil was measured after the experiment using callipers to be ${100}\,{\upmu \hbox {m}}\, \pm {5}\,{\upmu \hbox {m}}$.

### Ptychographic reconstructions: single polyimide foil

The 441 diffraction patterns were cropped to a size of $512 \times 512\,\text{ pixels }$ resulting in a pixel size of ${14.75}\,\hbox {nm}$ in the sample plane. The extended ptychographic iterative engine (ePIE)^38^ was used for the ptychographic single slice reconstruction. The update strength $\alpha$ for the object and the update strength $\beta$ for the probe were both set to 1.0 and the regularization parameter was set to 0.002. The initial object was chosen to be non-phase-shifting and non-absorbing. The initial probe was a gaussian with ${2}\,{\upmu \hbox {m}}$ FWHM and a phase curvature of ${-1}\,\hbox {mm}$. The reconstruction was run for 1000 iterations. The phase of the reconstructed object is shown in Fig. 3a.

The resolvable depth of field from the detector was calculated to be ${6.39}\,{\upmu \hbox {m}}$:

3 $$\begin{aligned} \text{ DOF}_{\text{ det }} = \frac{\lambda }{ \text{ NA}^2_{\text{ det }}} = \frac{4 \cdot \lambda d^2_{\text{ det }}}{ D^2_{\text{ det }}} = \frac{4 \cdot {0.136}\,\hbox {nm} \cdot \left( {4.16}\,{\hbox {m}} \right) ^2}{\left( {0.0384}\,\hbox {m} \right) ^2} = {6.39}\,{\upmu \hbox {m}}\,\,{,} \end{aligned}$$

where $\lambda$ is the X-ray photon wavelength, $\text {NA}_{\text{ det }}$ is the numerical aperture covered by the cropped detector images, $d_{\text{ det }}$ is the propagation distance from the sample to the detector, and $D_{\text{ det }}$ is the size of the cropped detector images. These ${6.39}\,{\upmu \hbox {m}}$ are smaller than the ${100}\,{\upmu \hbox {m}}$ separation of the copper(I) oxide cubes on the two sides of the polyimide tape, making the sample optically thick. Therefore, a multi-slice reconstruction was carried out using the 3PIE^21^ algorithm. As for the single slice reconstruction, the update strengths $\alpha$ for the object and $\beta$ for the probe where both set to 1.0 and the regularization was set to 0.002. Two object slices separated by ${100}\,{\upmu \hbox {m}}$ were used to model both sides of the polyimide tape. As there were no other windows in the beam and the polyimide foil itself is believed to be homogeneous, these two slices suffice to model the object. The propagation between these two slices was implemented as a convolution with a Fresnel kernel:

4 $$\begin{aligned} \text{ Prop}_{\Delta d_z} \left[ \Psi \left( {\mathbf {r}} \right) \right] = {\mathscr {F}}^{-1} \left\{ {\mathscr {F}} \left[ \Psi \left( {\mathbf {r}} \right) \right] \exp { \left( \text{ i } \pi \lambda \left( q_x^2 + q_y^2 \right) \cdot \Delta d_z \right) } \right\} \,\,{,} \end{aligned}$$

where $\Psi \left( {\mathbf {r}} \right)$ is the complex-valued wavefield to be propagated by the distance $\Delta d_z$ along the beam axis, ${\mathscr {F}}$ denotes the Fourier transform, ${\mathscr {F}}^{-1}$ denotes the inverse Fourier transform while $q_x$ and $q_y$ are the coordinates in Fourier space. The distance from the downstream object plane to the detector was kept the same. It fulfills the far-field condition, therefore this propagation was implemented as a simple Fourier transform, as in the ePIE algorithm.

The initial probe estimate was the same as for the previous ePIE reconstruction. Both object slices were initialized as non-absorbing and non-phase shifting. For the first 100 iterations, only the downstream sample slice was used, making the first 100 iterations identical to ePIE with particles from both sides in this slice. From the 101st iteration on the upstream slice was included in the update process and the particles on the side of the polyimide facing the source migrated into this slice. After 1000 iterations in total, no further changes in the reconstructed sample slices and the reconstructed probing wavefield could be observed. The reconstructed object slices are shown in Fig. 3b–d. Introducing a second sample slice and the propagation between the two slices into the algorithm, increased the number of computing tasks by a factor of three. Therefore, this reconstruction took about three times longer than the single-slice ePIE reconstruction.

As a confirmation, the initialization order of the two slices was reversed in an additional reconstruction: using only the upstream slice for the first 100 iterations and switching on the update of the downstream slice from iteration 101 on (see Fig. 5).

To check if the ${100}\,{\upmu \hbox {m}}$ separation of the two object slices was indeed correct, multiple reconstructions with varied distances were performed. We found that decreasing the slice separation resulted in the particles being reconstructed in both slices. Particles from the downstream slices were reconstructed weaker but also in the upstream slice and vice versa. Increasing the slice separation resulted in the over exaggeration in the particles in their respective slice and negative version of them in the other slice. Particles in the downstream slice appeared more phase shifting in the downstream slice and negatively phase shifting in the upstream slice. The same was true for the particles in the upstream slice. Most particles are either in the upstream slice or the downstream slice, and only in a very few cases particles from the two slices overlap in projection. Looking only at the majority of particles that appear only in either one of the slices, we could verify that indeed a slice separation of ${100}\,{\upmu \hbox {m}}$ created flat phases in the same position in the respective other slice. As the phase shifts of negative and mirror particles scaled with the change in distance of the object slices, we estimated the exact optical sample thickness to be ${100}\,{\upmu \hbox {m}} \pm {10}\,{\upmu \hbox {m}}$. Multiple ptychographic algorithms exists, which can estimate the slice separation by themselves^25,48^. Future experiments, in which the exact slice separation is unknown, can use those to perform the ptychographic multi-slice reconstructions.

### Sample preparation: chemical reactor

The preparation of the precursor solution was identical to the previous sample. Two reactions of particle growth were performed inside the chemical reactor. The chemical reactor was heated to ${150}\,^{\circ }\hbox {C}$ and kept at this temperature for ${4}\,\hbox {h}$ and ${12}\,\hbox {h}$ respectively. After cooling down and disassembling the chemical reactor, the windows were washed with ethanol prior to the SEM imaging. The SEM imaging proved that particles had grown on the inside surfaces of the windows in both runs. The chemical reactor was reassembled using the downstream window from the ${4}\,\hbox {h}$ reaction in the downstream position and the upstream window from the ${12}\,\hbox {h}$ reaction in the upstream position. Finally the chemical reactor was filled with benzyl alcohol before closing.

### Experiment: chemical reactor

The experiment was again performed at the hard X-ray nanoprobe station PtyNAMi of beamline P06 at the synchrotron radiation source PETRA III (Hamburg, Germany)^44,45^. The whole chemical reactor with all connections needed to potentially operated it, was placed inside the PtyNAMi setup^46,47^. In this experiment, the chemical reactor was kept at room temperature. The X-ray beam coming from the undulator was monochromatized to ${15.25}\,\hbox {keV}$ using a Si-(111) double-crystal monochromator. Higher harmonics were suppressed by a pair of flat horizontally deflecting mirrors. Nano-focussing lenses^42^ were used to focus the X-ray beam ${30}\,\hbox {mm}$ downstream of the most downstream lens exit. The sample was placed ${1.5}\,\hbox {mm}$ downstream of the X-ray beam focus, resulting in a beam size of about ${450}\,\hbox {nm}$ (FWHM) on the sample. The depth of field was calculated to be:

5 $$\begin{aligned} \text{ DOF}_{\text{ NFL }} = \frac{\lambda }{\text {NA}^2_{\text{ NFL }}} = \frac{4 \cdot \lambda f^2_{\text{ NFL }}}{ D^2_{\text{ NFL }}} = \frac{4 \cdot {0.081}\,\hbox {nm} \cdot \left( {30}\,\hbox {mm}\right) ^2}{\left( {0.04}\,\hbox {mm}\right) ^2} = {182.95}\,{\upmu \hbox {m}} \end{aligned}$$

Piezoelectric motors were used to scan the sample on a rectangular grid with a size of ${8}\,{\upmu \hbox {m}} \times {8}\,{\upmu \hbox {m}}$ in $80 \times 80$ steps. At each of the $81 \times 81 = 6\,561$ positions, a far-field diffraction pattern was recorded with ${20}\,\hbox {ms}$ exposure time using an EIGER X 4M detector (DECTRIS, Switzerland, ${75}\,{\upmu \hbox {m}}$ pixel size) positioned ${3.435}\,\hbox {m}$ downstream of the sample. The relative sample positions were measured by three interferometers retro-reflected by a ball-lens located below the sample^46^. Using again a cropping of $512 \times 512\,\text{ pixels }$, the minimal possible depth of field for this experiment was calcualted to be:

6 $$\begin{aligned} \text{ DOF}_{\text{ det }} = \frac{\lambda }{ \text {NA}^2_{\text{ det }}} = \frac{4 \cdot \lambda d^2_{\text{ det }}}{ D^2_{\text{ det }}} = \frac{4 \cdot {0.085}\,\hbox {nm} \cdot \left( {3.435}\,\hbox {m} \right) ^2}{\left( {0.0384}\,\hbox {m} \right) ^2} = {2.58}\,{\upmu \hbox {m}}\,\,{,} \end{aligned}$$

Again the whole sample is much thicker than this, but the copper(I) oxide nano cubes themselves fulfil the thin sample approximation, even if the signal were to scatter sufficiently over the whole size of the cropped diffraction patterns. The distance between the inside surfaces of the upstream window and the downstream window was estimated to be ${650}\,{\upmu \hbox {m}}$. Both windows had a slight curvature, most likely due to expansion while the chemical reactor was heated during the sample growth. Hence the exact distance also depended on the location of the field of view on the window and might also change while heating the chemical reactor.

### Ptychographic reconstructions: chemical reactor

The $6\,561$ recorded diffraction patterns were cropped to a size of $512 \times 512\,\text{ pixels }$ resulting in a pixel size of ${7.27}\,\hbox {nm}$ in the sample plane. The extended ptychographic iterative engine (ePIE)^38^ was used for the ptychographic single slice reconstruction. The update strength $\alpha$ for the object and the update strength $\beta$ for the probe were both set to 1.0 and the regularization parameter was set to 0.002. The initial object was chosen to be non-phase-shifting and non-absorbing. The initial probe was a gaussian with ${500}\,\hbox {nm}$ FWHM and a phase curvature of ${+2}\,\hbox {mm}$. The reconstruction was run for 1000 iterations. The phase of the reconstructed object is shown in Fig. 6b.

The multi-slice reconstruction using the 3PIE algorithm was performed as before. The samples was modeled by two slices, separated by ${650}\,{\upmu \hbox {m}}$ of free propagation space. Both object slices were initalized as non-absorbing and non-phase shifting. The inital estimate for the probing beam was the same as for the single slice reconstruction using the ePIE algorithm. For the first 100 iterations, only the upstream sample slice was used, making the first 100 iterations identical to ePIE with particles from windows in this slice. From the 101st iteration on the downstream slice was included in the update process and the particles on the downstream window migrated into this slice. After 1000 iterations in total, no further changes in the reconstructed sample slices and the reconstructed probing wavefield could be observed. The phases of the reconstructed object slices are shown in Fig. 6b–d.

## Data Availability

The raw dataset^49^ used for the results presented in this article is openly available via zenodo.
